# Comparison of clinical and dental panoramic findings: a practice-based crossover study

**DOI:** 10.1186/1472-6831-13-48

**Published:** 2013-09-26

**Authors:** Marc A Moll, Miriam Seuthe, Constantin von See, Antonia Zapf, Else Hornecker, Rainer F Mausberg, Dirk Ziebolz

**Affiliations:** 1 Department of Preventive Dentistry, Periodontology and Cariology, University Medical Centre, Robert-Koch Str. 40, Goettingen D-37075, Germany; 2 Department of Oral and Maxillofacial Surgery, Hanover Medical School, Hanover, Germany; 3 Dental office, German Armed Forces, Munster, Germany; 4 Department of Medical Statistics, Hanover Medical School, Hanover, Germany

**Keywords:** Dental findings, Radiographic findings, Dental panoramic radiography (DPR), Clinical examination

## Abstract

**Background:**

Aim was to compare clinical findings with x-ray findings using dental panoramic radiography (DPR). In addition, type and frequency of secondary findings in x-rays were investigated.

**Methods:**

Patients were selected on the basis of available DPRs (not older than 12 months). No therapeutic measures were permitted between the DPR and the clinical findings. The clinical findings were carried out by several investigators who had no knowledge of the purpose of the study. A calibrated investigator established the x-ray findings, independently and without prior knowledge of the clinical findings. The evaluation parameters for each tooth were: missing, healthy, carious, restorative or prosthetically sufficient or insufficient treatment. Type and frequency of additional findings in the DPR were documented, e.g. quality of a root canal filling and apical changes.

**Results:**

Findings of 275 patients were available. Comparison showed a correspondence between clinical and radiographic finding in 93.6% of all teeth (n = 7,789). The differences were not significant (p > 0.05). Regarding carious as well as insufficiently restored or prosthetically treated teeth, respectively there were significant differences between the two methods (p < 0.05). The DPRs showed additional findings: root fillings in 259 teeth and 145 teeth with periapical changes.

**Conclusions:**

With reference to the assessment of teeth, there was no difference between the two methods. However, in the evaluation of carious as well as teeth with insufficiently restorative or prosthetic treatment, there was a clear discrepancy between the two methods. Therefore, it would have been possible to have dispensed with x-rays. Nevertheless, additional x-ray findings were found.

## Background

Precise dental ascertainment of findings is the key to achieving adequate diagnostics, as well as the therapy based on this and the best possible treatment of the patient. In addition to a comprehensive anamnesis, the documentation of extra- and intra-oral clinical findings is necessary. The clinical dental examination includes, in addition to assessment of the mucous membranes, the condition of the teeth (healthy or carious), the restorative and prosthetic treatment of the teeth (sufficient or insufficient), as well as sensitivity testing and determination of the periodontal situation [1].

In order to complete the dental findings x-rays are recommended [2]. Dental panoramic radiography (DPR) provides an overview, and represents a sensible and frequently used radiological basis; it enables an assessment of the hard tissue structures of the facial area [3]. In this way, clinical findings can be verified and supplemented by important information. At the same time, however, the value should be greater than the potential risk of genotoxic effects caused by x-rays [2,4]; in this connection, the quality of the x-rays is of great importance [5].

In Germany, the relevance of DPR in providing an overview is undisputed [6]. Already in the 1970s, the routine preparation of a DPR in dental practices was promoted for the pre-treatment examination: It enables the early diagnosis of tooth and jaw anomalies [7], and the treatment costs may be reduced in the long-term [8]. Rushton and Horner (1996), in contrast, have questioned the importance of x-ray findings for dental diagnostics in the routine recording of clinical dental findings [9]. They argue that the differences between tactile-visual findings and x-ray findings are too small and may cause an unnecessary exposure of the patient to x-rays [9]. Moreover, the diagnostic precision has been questioned by some other authors [10,11]. The European Guideline on Radiation in Dental Radiography Issue No. 136 says that in adult patients DPR may be indicated in a limited number of dental problems [12].

Up until now, the question of the extent to which supplementary x-rays provide additional and valuable information during the clinical dental examination has not been unambiguously clarified. Thus, it is conceivable that, based on a proficient, reliable acquisition of clinical findings during the first (check-up) examination in everyday practice, the sense of an additional DPR is questionable. Accordingly, it is possible that either no x-rays are taken or, if it is, only an inadequate assessment will take place instead of obtaining detailed x-ray findings. However, the consequence may be the lack of or inadequate diagnostics and possibly inadequate therapy. In particular problems, i.e. suspicion on carious lesions and periapicale problems bitewing radiographs and/or periapicale radiographs, respectively, are the method of choice [13]. Nevertheless, DPRs nowadays are commonly used. The reason may be that additional findings can be detected; however, this does not justify routinely preparing DPRs. For this reason, it seems appropriate to systematically examine the question of the additional value of DPR in the context of the first dental (check-up) examination.

The aim of this study was to compare the clinical dental findings of the first (check-up) examination in dental practices with currently available DPRs in terms of an assessment of healthy or carious, as well as restorative and prosthetically sufficient or insufficiently treated teeth. Also, additional x-ray findings were investigated, in particular, sufficient and insufficient root canal fillings, teeth with apicectomy or periapical changes, impacted teeth, as well as shadowing of the maxillary sinus.

The following hypotheses were formulated: clinical findings and x-ray findings based on DPR show only minor assessment differences. However, DPR enables the detection of a number of additional information which is needed for comprehensive diagnostics.

## Methods

The study was a cross-sectional investigation based on available patients’ records (clinical findings and x-ray findings (DPR)) from three dental practices of the German Forces (locations: Wilhelmshaven (navy), Munster (army) and Köln-Wahn (air-force).^a^ Authorization for carrying out the study was obtained from the Bundesministerium für Verteidigung (German Ministry of Defense), Fü San I/1, (File NO.: 42-13-05 dated 18.07.06).

### Participants/x-rays

Making use of the x-ray control books of the participating centers, all DPRs from soldiers dating back to the years 2007/2008 were determined. The x-rays had to have been prepared within a maximum of 12 months before the dental clinical (check-up) examination. No additional DPR was prepared for the study.

The following inclusion criteria were defined: current and evaluable DPR and complete clinical dental finding, no therapeutic measures having taken place between preparation of the DPR and documenting of the clinical finding. Only dental records of male soldiers who had signed up for a fixed period (minimally 4 years) or professional soldiers were included. The selection of the DPRs was carried out in accordance with the inclusion and exclusion criteria, respectively, shown in Table 1. Furthermore, subjects with removable dental restoration were excluded.

**Table 1:** Quality criteria for the x-rays (DPR) in the study

**Inclusion criteria**	**Exclusion criteria**
• DPR at the time of the study not older than 12 months	• Metallic foreign bodies in the head region, e.g. earrings or piercings
• Standardized setting of the x-ray equipment: chin support, bite bar, voltage (70 kV) and current (9 mA)	• Asymmetrical positioning of the patient
	• False positioning of the patient’s head
	• Improper presentation of the jaws and the teeth
• Comparable/good image quality: (no film overexposure and no soiling of the film)	• recognizable movement of the patient while the picture was being taken
• Presentation of jaws and teeth distortion-free as much as possible	• overlaying effects

### Clinical examination

Clinical findings (visual-tactile) were recorded on one occasion under standardized conditions (mirror, dental probe, illumination) during a routine first (check-up) examination in those dental centers participating in the study. The findings were randomly taken from two dentists per dental center, all dentists were skilled in dental examination; they were not calibrated and had no knowledge in the study. At the time of the clinical examination, the radiographic findings of the soldiers were not known to the six dentists.

The following parameters were recorded: missing teeth, healthy and carious teeth, sufficient and insufficient fillings (amalgam, composite, inlay), as well as prosthetic treatment (crowns/partial crowns), combination of findings: filling or restoration with caries in the sense of a primary or secondary caries, as well as implant treatment. Because of irregular and imprecise details or information, respectively, the sensitivity test, initial caries lesions and the periodontal situation were not taken into account.

### Radiographic examination

The findings of the DPRs were taken by a sole investigator (MS) who was calibrated in advance (Kappa value ≥ 0.8).

The assessment of the DPRs was carried out under standardized conditions in a shaded room with an x-ray film viewer (light box) capable of functioning under such conditions; the clinical findings were not known to this investigator. The inclusion and exclusion criteria for the panoramic radiographs are given in Table 1. Each DPR was assessed twice at a 3-week interval. In the case of deviation between the two assessments, the final finding was established by a third assessment.

For the assessment of the x-rays, the same parameters as for the clinical findings were used. Fillings and restorations were judged to be sufficient if there was a smooth transition between restoration and tooth. Overhanging fillings and crown margins, as well as radiolucent gaps between restoration and tooth indicating deficient marginal fit in the sense of a secondary caries were judged to be insufficient. Implants were simply registered without assessment.

In addition, various additional findings were recorded:

*Endodontic findings*: proper root canal filling, deficient root canal filling based on the following criteria: insufficiently filled or overfilled, coronal leakage, improper homogeneity of the filling material.

*Periapical region*: periodontal ligament space widened, periapical change, apical radiolucency, root-tip-resection.

*Further findings*: impacted and ectopic tooth, root residue, shadowing of the maxillary sinus.

### Statistical analysis

Statistical analysis was performed with the commercially available program SPSS 14.0 (SPSS, Inc., Chicago, IL, USA). The statistical comparison of the clinical and radiological findings was made using the paired-rank test. The level of significance was set at p < 0.05.

## Results

### Participants

A total of 302 patients’ records were available for the period under investigation. According to the inclusion criteria for the x-rays 275 patients’ records could be examined and analyzed. The age of the subjects ranged from 25 to 35 years.

### Clinical examination

The results of the clinical findings are given in Table 2.

**Table 2:** Summary of the dental findings and x-ray findings, as well as the number of agreements

*Parameter for findings	**Clinical findings (n = 8800)/[%])**	**X-ray findings (n = 8800/[%])**	**Number of accordance [%]**	**Significance level (p value)**
**Missing teeth**	1007 (11.4)	1011 (11.5)	996 (99.6)	n.s.
**Healthy teeth**	4650 (52.8)	4858 (55.2)	4587 (95.7)	n.s.
**Carious teeth**	318 (3.61)	212 (2.41)	176 (66.6)	p < 0.05
**Tooth sufficiently filled**	1978 (22.5)	2009 (22.8)	1873 (98.5)	n.s.
**Tooth insufficiently filled**	125 (1.42)	116 (1.32)	71 (92.8)	p < 0.05
**Tooth sufficiently prosthetically treated**	446 (5.07)	429 (4.88)	425 (96.2)	n.s.
**Tooth insufficiently prosthetically treated**	3 (0.003)	16 (0.018)	3 (18.8)	p < 0.05
**Implant**	16 (0.018)	16 (0.018)	16 (100.0)	n.s.
**Combination (restoration and carious)**	136 (1.55)	133 (1.51)	91 (97.8)	n.s.
**Clinically: suspected caries**	121 (1.38)	--	--	--

(*Parameter for findings: in accordance with Material and Methods, n.s.: not significant = p > 0.05).

Acting on the assumption of 32 teeth in the permanent dentition per participant, in total there were maximally 8,800 teeth to assess, of these 1,007 teeth were clinically lacking, 553 of these were wisdom teeth. 4,650 teeth were classed as being healthy (52.8%), and 318 teeth showed carious lesions (3.6%). 2,424 teeth (27.5%) showed sufficient fillings (n = 1978) or sufficient prosthetic restoration (n = 446). In contrast, 128 teeth (1.5%) were treated insufficiently (restorative: n = 125, prosthetically: n = 3). 136 (1.5%) teeth showed fillings or restorations as well as caries lesions. In the case of 121 teeth (1.38%), the clinical examination revealed “suspected caries”: 16 teeth had been replaced by an implant.

### Radiographic examination

The results of the radiological findings are shown in Table 2.

Of 8,800 maximally possible teeth, 1,011 teeth (11.5%) were missing; of which 553 were wisdom teeth. More than 50% of the teeth were considered to be healthy (n = 4,858). With reference to the x-ray findings carious lesions were found in 212 teeth (2.4%). 2,009 teeth were sufficiently treated with fillings (22.8%) and 429 prosthetically (4.9%). In contrast, 132 (1.5%) teeth showed insufficiently restorative (n = 116) or prosthetical (n = 16) treatment. 133 (1.5%) teeth had fillings or restorations as well as caries. In total, 16 implants were present.

Details of additional findings are summarized in Table 3.

**Table 3:** Number of additional findings (n) in the x-rays (DPR)

**Type of additional findings**			**Number of additional findings [%]**
**Endodontic (n = 8800)**	**Total**		**259 (2.9%)**
	sufficient		184 (71%)
	insufficient	1 insufficiently filled	42 (16%)
		2 insufficient homogeneity	2 (1%)
		3 over-filled	5 (2%)
		4 coronal leakage	11 (4%)
		combination of 1 - 4	15 (6%)
**Periapical region (n = 8800)**	**Total**		**145 (1.7%)**
	periodontal ligament space widened		54 (37%)
	periapical change		4 (3%)
	apical raduiolucency	teeth with RCF	72 (50%)
		teeth without RCF	15 (10%)
**Other (n = 8800)**	**Total**		**171 (1.9%)**
	impacted or ectopic teeth		132 (77%)
	root residue		3 (2%)
	root-tip-resection		31 (18%)
	shadowing of the maxillary sinus		5 (3%)

(RCF: root canal filling).

*Endodontic findings and assessment of the periapical region*: 259 of all teeth evaluated (2.9%) showed root canal fillings; in accordance with the laid-down criteria, of these teeth 184 (71.0%) were assessed to have sufficient endodontic treatment. In the case of the root canal fillings considered to be insufficient. (n = 75), the majority (n = 42, 56%) showed inadequately filled canals. Independent of endodontic treatment and/or the quality of the existing root canal filling, in total, 145 teeth showed periapical changes (1.6%). About half of them (n = 72; 0.8%) were root-canal-filled teeth.

*Further findings*: In total, it was possible to establish 171 additional abnormalities/findings: 132 impacted or ectopic wisdom teeth (79.5%), 31 teeth were root-tip resected (18.7%), and 3 root residues (1.8%) were found. In five cases, the maxillary sinus showed shadowing (3%).

### Comparison of clinical and radiographic findings

It was possible to establish agreement in 93.6% of cases of all parameters between clinical and radiological findings (Table 4). There was no significant difference between the two methods of assessment (p > 0.05).

**Table 4:** Comparison between clinical (C) and radiological findings (R) using the paired-rank-test

	**C**	**0**	**1**	**2**	**3**	**4**	**5**	**6**	**7**	**8**	**9**	**Total**
**R**												
**0**		**996**	7	4	0	0	4	0	0	0	0	1011
		**11.3%**	0.08%	0.05%	0.00%	0.00%	0.05%	0.00%	0.00%	0.00%	0.00%	11.5%
**1**		7	**4587**	130	18	2	0	0	0	3	111	4858
		0.08%	**52.1%**	1.48%	0.20%	0.02%	0.00%	0.00%	0.00%	0.03%	1.26%	55.2%
**2**		0	23	**176**	5	1	0	0	0	0	7	212
		0.00%	0.26%	**2.0%**	0.06%	0.01%	0.00%	0.00%	0.00%	0.00%	0.08%	2.41%
**3**		1	29	5	**1873**	51	6	0	0	41	3	2009
		0.01%	0.33%	0.06%	**21.3%**	0.58%	0.07%	0.00%	0.00%	0.47%	0.03%	22.8%
**4**		0	2	0	43	**71**	0	0	0	0	0	116
		0.00%	0.02%	0.00%	0.49%	**0.81%**	0.00%	0.00%	0.00%	0.00%	0.00%	1.32%
**5**		1	0	0	2	0	**425**	0	0	1	0	429
		0.01%	0.00%	0.00%	0.02%	0.00%	**4.83%**	0.00%	0.00%	0.01%	0.00%	4.88%
**6**		1	0	0	1	0	11	**3**	0	0	0	16
		0.01%	0.00%	0.00%	0.01%	0.00%	0.13%	**0.03%**	0.00%	0.00%	0.00%	0.18%
**7**		0	0	0	0	0	0	0	**16**	0	0	16
		0.00%	0.00%	0.00%	0.00%	0.00%	0.00%	0.00%	**0.18%**	0.00%	0.00%	0.18%
**8**		1	2	3	36	0	0	0	0	**91**	0	133
		0.01%	0.02%	0.03%	0.41%	0.00%	0.00%	0.00%	0.00%	**1.03%**	0.00%	1.51%
**Total**		1007	4650	318	1978	125	446	3	16	136	121	8800
		11.4%	52.8%	3.61%	22.5%	1.42%	5.07%	0.03%	0.18%	1.55%	1.38%	100%

(0 = tooth missing, 1 = healthy, 2 = caries, 3 = filling sufficient, 4 = filling insufficient, 5 = prosthetics sufficient, 6 = prosthetics insufficient, 7 = implant, 8 = combination of 2 and 3-6, 9 = suspected caries; number of accordance).

In 6.4% of the teeth discrepancies between the two method were found, and these were distributed mainly regarding the following findings: caries and insufficient restorative or prosthetic treatment (Tables 2 and 4). Within this group, there were significant differences between clinical findings and x-ray findings (p < 0.05; Table 2). Clinically, there were 130 carious lesions present that could not be established by the x-ray findings. In contrast, 23 lesions in the sense of a carious lesion were only identified by means of DPR findings. In the assessment of the existing restorations, 54 teeth showed a clinically sufficient filling (n = 43) or sufficient prosthetic treatment (n = 11) that were judged with reference to x-rays to be insufficient. This contrasted with 51 clinically insufficient fillings that were assessed by x-rays to be sufficient.

For the other parameters (healthy tooth, filling insufficient, prosthetic sufficient), there was no significant difference between the two methods of assessment (p > 0.05; Table 2).

## Discussion

The aim of the study was to evaluate the extent to which clinical findings and x-ray findings based on dental panoramic radiography (DPR) correspond with and differ from one another.

### Summary of the main results

With an agreement of 93.6%, the results showed no significant differences between the two investigative methods (p > 0.05). In the assessment of “caries”, as well as insufficient fillings and prosthetic restorations, there were, however, to some extent, substantial differences between clinical and radiological findings. In addition, the x-rays revealed a number of additional findings: endodontic findings (n = 259), changes in the periapical region (n = 145) and others (n = 171), such as for example impacted or ectopic teeth and shadowing of the maxillary sinus.

### Interpretation of the results and comparison with the international literature

The high level of agreement of both methods of assessment according to the criteria for evaluation laid down previously is noteworthy and once more questions the preparation of a DPR as a supplementary diagnostic tool in the context of a comprehensive dental examination. Accordingly, in a large number of cases, it would have been possible to have dispensed with x-rays. Only in the case of 6.4% of teeth there was no agreement between the two methods of investigation. Other studies have also raised the same question [14,15].

#### Assessment of healthy and carious teeth

In this study, the parameter “carious teeth” showed a significant difference (p < 0.05) between clinical and x-ray findings. Therefore, the sensitivity for carious teeth is lower then for healthy teeth (healthy: 95.7%, carious: 66.6%). In contrast to the study of Valachovic et al. (1986), we obtained better results with regard to the agreement between clinical and x-ray findings in the assessment of healthy and carious teeth. For carious teeth, those authors found with 54%, only a small sensitivity (agreement) of the x-ray findings with the clinical findings [10]. Even more unfavorable were the results of Moleander et al. (1993) with 47% [11]. One suspects that the better results in the current study can be traced back to the technical advances in dental radiology. Particularly for the detection of interproximal caries lesions, nowadays x-rays, DPRs and specially bitewings, are considered to be the most suitable diagnostic aid. However, the prerequisite for this is that, in the preparation of the x-rays, no errors due to the method or production occur [16] and that the x-rays show no deficiencies in quality [5]. Certainly, bitewings are the radiographic method indicated to assess interproximal carious lesions [17]. According to Rushton et al. (2002) bitewing radiographs will reduce the diagnostic yield identified solely by DPR [18]. Therefore, DPRs are unsuitable for carious diagnostic. The finding “carious lesion” showed only in 66.6% of cases an agreement between the two investigative methods. Douglass et al. (1986) found comparable results: in 60% of cases certain recognition was achieved with a combination of DPR and bitewing radiographs [19]. Two recent studies in which, in addition, one or more investigators were calibrated, showed a comparably high agreement in the identification of carious lesions [20,21]. The values obtained by Hopcraft and Morgan (2005) using x-rays were, for interproximal caries recognition, around 93% to 97% and for occlusal carious lesions 33% to 42%; however, in addition to DPR, bitewing radiographs were also included in this study [20]. In the study presented here, more carious lesions were found in the clinical examination than in the x-ray findings. This result supports earlier investigations in which, likewise, the clinical finding was superior to the findings based on DPR [22-24]. In contrast, Heners and Riotte (1978) found more carious lesions using bitewing radiographs than in the clinical examination [25]. Therefore, the results of this study can not be compared with the results of our study. Deductive, the results of our study shows that DPR is not a good diagnostic tool to detect carious lesions. For revealing carious lesions Rushton et al. (2002) prefer posterior bitewing radiographs [18].

#### Assessment of the restorations

Overall, the differences between the two methods in the assessment of “insufficient filling” and “insufficient prosthetic restoration” were greater than the agreement. To the best of our knowledge, there have been no data available in the literature, up until now, regarding this question.

The extent to which the margin fit can be evaluated in x-rays is considered to be an important criterion for deciding whether there is sufficient or insufficient dental provision. The evaluation of the various possibilities of deficient/defective margin fit was carried out in the present study only in terms of “sufficient” or “insufficient”. Material investigations have drawn attention to the fact that an adequate difference in opacity between restoration material (composite) and dental enamel must exist in order to assess quality of the filling [26]. For this reason, it may be possible that in the present study imprecise statements about the margin fit were mostly found in the case of composite restorations. Moreover, it should be considered that, because of the fuzziness in the DPR, the margins could not always be unambiguously evaluated. These points could explain the discrepancy between the two investigative methods in relation to the assessment of “insufficient filling”, whereby in 92.8% of teeth an agreement could be found (Table 2). In contrast to the assessment of fillings, “insufficient prosthetic” was more frequently made in the x-rays than clinically. A possible explanation for this could be that a clinical, visual-tactile judgement of the frequently sub-gingival located crown margins is possibly more difficult. Accordingly, in routine dental health check of adult patients DPR is not necceassary. This is in accord with other authors [13-15,18].

Consequently, the hypothesis formulated in advance, that clinical and radiological findings show only small differences, was confirmed. At the same time, pathological findings (caries, insufficient filling) are more frequently uncovered clinically than by using x-rays. Whereas material properties of prosthetic restorations (e.g. precious metals, ceramics) have been well investigated, there is a lack of scientific data regarding the quality of provision of fixed artificial dentition from clinical practice; compared to other forms of artificial dentition, however, it was possible to record high probabilities of survival [27,28]. Taking into account the results presented here, the quality of fixed artificial dentition and the possibility of their being assessable in x-rays appears to be subject to limitations; it is possible, that because of the two-dimensional presentation in x-rays the number of additional findings could have been overestimated.

#### Additional findings

The assessment of the DPR revealed a substantial number of additional clinically non-conspicuous findings (Table 4). It is here that the strength of the DPR as a supplementary diagnostic aid to the clinical findings lies as, in this way, an additional/substantial gain in information can be achieved. At the same time, the advantages and disadvantages in the preparation of a DPR in the context of a risk/benefit analysis should be well weighed up.

In this study, because DPR provides an overview, it was possible to obtain in total 171 additional findings. Compared with other studies, that is certainly a low number, but the distribution in relation to the corresponding findings (impacted teeth, teeth with root-tip-resection etc.) is, however, very similar when frequencies are compared [29,30]. Also, the association between periapical condition and sufficient root filling has already been discussed in some studies [31,32] and is confirmed by the results of this investigation. Accordingly, the second hypothesis – that by using DPR a large number of additional findings can be uncovered – was also confirmed. However, the quantity of additional findings nonetheless makes the DPR an important aid in dental diagnostics. Therefore, the preparation of a DPR including meticulous x-ray findings in the context of a first dental (check-up) examination can be a helpful measure. In contrast, Rushton and Rushton (2012) in comparing 740 DPR and intraoral radiographs showed, that only in 32 DPR additional information were found [13]. The authors conclude that there is no reason for the use for DPRs in routine dental health examinations [13]. The European Guideline on Radiation in Dental Radiography Issue No. 136 supports this opinion [12].

### Implication for the practice

The results presented in this study indicate that using comprehensive and good clinical dental diagnostics for the assessment of healthy/carious and/or sufficiently/insufficiently restored and prosthetically treated teeth, routine DPR can be dispensed with. However, because of the additional and unintended findings, for correct diagnosis and adequate therapy, the combination of clinical findings and x-ray findings is sensible.

### Strengths and limitations of the study

The investigation presented here is a practice-based clinical study. Overall, there is a lack of practice-related studies, thus, this study provides a scientific contribution from daily practice.

The clinical findings were obtained under conditions to be found in a dental practice and were undertaken by a number of experienced investigators/skilled dentists without prior calibration and knowledge of the participants regarding the study. In this way, a study-determined effect can be excluded. However, the fact needs to be taken into account that differences may have occurred as a result of individual subjectivity of the examining dentists, different levels of care and clinical experience in the recording of data. Overall, however, a high quality standard appears to predominate in the investigation as, despite the absence of calibration of the clinical investigators, there was a high level of agreement with the x-ray findings. In contrast to this, the x-ray evaluation was carried out with a high standard following prior investigator calibration. This should ensure that the x-ray findings were recorded completely and comprehensively, with the aim of establishing comparability and in order to assess the additional value of the DPR. Moreover, because of the large number of teeth investigated and strict selection criteria, the results can be viewed as being representative.

In the context of the clinical dental examination, only sporadically sensitivity tests were undertaken, so that these could not be included in the study. It is therefore to be assumed that in making the comparison with the x-ray findings, additional agreement with endodontic and periapical findings could arise.

Furthermore, the assessment of periodontal bone level was dispensed, because of the young age of the study population (25-35 years). In addition, in a first dental (check up) examination the PSR®/PSI is essential for initial periodontal examination [33]. This finding was not available in all cases. Furthermore, Ziebolz et al. (2011) showed, that DPR is of no value in cases of initial periodontal problems [33].

## Conclusions

There was no difference between clinical and x-ray findings. However, with regard to the assessment of carious as well as insufficiently filled or prosthetically treated teeth, there was a clear discrepancy between the two methods of investigation. At the same time, DPR findings were inferior to clinical findings in relation to the parameters “carious tooth” and “insufficient filling”. Accordingly, the DPR gives the clinician no additional gain in information in this area. Therefore, it would have been possible to have dispensed with x-rays. Nevertheless, additional x-ray findings were found.

## Endnote

^a^In the German Forces, before each deployment abroad, dental findings are documented and x-ray findings recorded based on up-to-date dental panoramic radiography (DPR).

## Competing interests

The authors declare that they have no financial or other relationships that might lead to competing interests.

## Authors’ contributions

MM interpreted the data and wrote the manuscript. MS carried out the radiological examination and performed the determination of the study data. CvS has been involved in revising the manuscript critically for important intellectual content and has given final approval of the version to be published. AZ performed the statistical analysis. EH conceived of the study, and participated in its design, and has been involved in drafting the manuscript. RM has made substantial contributions to conception and design of the study, DZ was the head of the study and has made substantial contributions to conception, design and coordination. All authors read and approved the final manuscript.

## Pre-publication history

The pre-publication history for this paper can be accessed here:

http://www.biomedcentral.com/1472-6831/13/48/prepub
